# Altered large-scale brain network interactions associated with HIV infection and error processing

**DOI:** 10.1162/netn_a_00241

**Published:** 2022-07-01

**Authors:** Jessica S. Flannery, Michael C. Riedel, Lauren D. Hill-Bowen, Ranjita Poudel, Katherine L. Bottenhorn, Taylor Salo, Angela R. Laird, Raul Gonzalez, Matthew T. Sutherland

**Affiliations:** Department of Psychology and Neuroscience, University of North Carolina at Chapel Hill, Chapel Hill, NC, USA; Department of Physics, Florida International University, Miami, FL, USA; Department of Psychology, Florida International University, Miami, FL, USA; Department of Population and Public Health Sciences, University of Southern California, Los Angeles, CA, USA

**Keywords:** HIV, Resting-state functional connectivity, Error awareness, Cannabis, Default mode network, Central executive network, Salience network

## Abstract

Altered activity within and between large-scale brain networks has been implicated across various neuropsychiatric conditions. However, patterns of network dysregulation associated with human immunodeficiency virus (HIV), and further impacted by cannabis (CB) use, remain to be delineated. We examined the impact of HIV and CB on resting-state functional connectivity (rsFC) between brain networks and associations with error awareness and error-related network responsivity. Participants (*N* = 106), stratified into four groups (HIV+/CB+, HIV+/CB−, HIV−/CB+, HIV−/CB−), underwent fMRI scanning while completing a resting-state scan and a modified Go/NoGo paradigm assessing brain responsivity to errors and explicit error awareness. We examined separate and interactive effects of HIV and CB on resource allocation indexes (RAIs), a measure quantifying rsFC strength between the default mode network (DMN), central executive network (CEN), and salience network (SN). We observed reduced RAIs among HIV+ (vs. HIV−) participants, which was driven by *increased* SN-DMN rsFC. No group differences were detected for SN-CEN rsFC. Increased SN-DMN rsFC correlated with diminished error awareness, but not with error-related network responsivity. These outcomes highlight altered network interactions among participants with HIV and suggest such rsFC dysregulation may persist during task performance, reflecting an inability to disengage irrelevant mental operations, ultimately hindering error processing.

## Author Summary

One systems-level framework proposes that dysregulated brain network interactions may underlie neurocognitive aspects of addiction, neurodegeneration, and other neuropsychiatric conditions. Three specific networks have been highlighted: the default mode network (DMN), the central executive network (CEN), and the salience network (SN). To examine alterations linked with human immunodeficiency virus (HIV) and cannabis (CB) use across these three canonical brain networks, we utilized a composite network association index, previously used to assess drug-related effects on brain networks. This study provides a novel perspective on dysregulated DMN and SN rsFC among participants living with HIV and delineates potential implications for error-monitoring behavior.

## INTRODUCTION

The human immunodeficiency virus (HIV) is a chronic infection that dysregulates the immune system and enters the central nervous system (Williams et al., 2020). Neuroinflammation due to HIV, combined with the additive and/or interactive effects of opportunistic infections, aging, and substance use, contributes to neurocognitive impairments that persist in the antiretroviral therapy era (Gao et al., 2020; Hong & Banks, 2015; Rich et al., 2020). In particular, the impact of cannabis (CB) use in the context of HIV infection may be especially relevant when considering neurocognitive function given the prevalence of use among people living with HIV (PLWH; D’Souza et al., 2012; Montgomery et al., 2019; Pacek et al., 2018; Rizzo et al., 2018) and frequent reports of CB use to relieve HIV-related symptoms (Harris et al., 2014; Towe et al., 2020). As neurocognitive impairments among PLWH negatively impact disease management, employment, and quality of life (Blackstone et al., 2012; Cattie et al., 2012), there is a need to more fully characterize neurobiological contributors to such symptoms (Robertson et al., 2020; Williams et al., 2020).

One systems-level model proposes that the neurobiological underpinnings of many neuropsychiatric conditions can be conceptualized in terms of dysregulated interactions between large-scale brain networks (B. Menon, 2019; V. Menon, 2011). This framework has proved useful for understanding altered brain function associated with addiction (Bednarski et al., 2011; Sutherland et al., 2012; R. Zhang & Volkow, 2019), Alzheimer’s disease (AD; Damoiseaux et al., 2012; Jones et al., 2016), attention deficit hyperactivity disorder (ADHD; Liddle et al., 2011; Peterson et al., 2009), frontotemporal dementia, mood disorders (Bartova et al., 2015), and schizophrenia (Zhou et al., 2016). The three widely recognized brain networks implicated in this model are the default mode network (DMN), the central executive network (CEN), and the salience network (SN; e.g., V. Menon, 2011; Moradi et al., 2020; Sutherland et al., 2012). The DMN is primarily engaged during intrinsic thought processes that arise independent of external stimuli and is thought to be involved with internal and self-referential information processing (Buckner & DiNicola, 2019; Di & Biswal, 2014; Moradi et al., 2020), whereas the CEN is engaged during tasks demanding attention and cognitive control and is thought to be involved with processing external stimuli (Seeley et al., 2007; Sridharan et al., 2008). DMN and CEN activity are generally anticorrelated, and the SN is thought to toggle neurocognitive resources between these two networks, thereby prioritizing processing of the currently most pertinent information (Chong et al., 2017; V. Menon, 2011; Menon & Uddin, 2010).

The resource allocation index (RAI) is a previously developed metric quantifying network-level interactions based on the theorized role of the SN in toggling activity between the DMN and CEN such that higher RAI values indicate increased synchrony between the SN and CEN and/or decreased synchrony between the SN and DMN (Lerman et al., 2014). The RAI has been used to evaluate dysfunction in this relative balance between SN-DMN and SN-CEN coupling (Choi et al., 2013; Lerman et al., 2014; Reese et al., 2019; J. T. Zhang et al., 2017). For example, the attentional and cognitive deficits characterizing nicotine withdrawal have been linked to reduced activation in brain regions comprising the CEN and less suppression of activity in regions comprising the DMN (Falcone et al., 2014; Hahn et al., 2007; Lerman et al., 2014; Loughead et al., 2010; Tanabe et al., 2011). These observations support the hypothesis that the SN may allocate attentional resources toward nicotine withdrawal–related processes (e.g., craving), thereby biasing activity toward the DMN and away from the CEN (Sutherland et al., 2012; Sutherland & Stein, 2018; Sutherland et al., 2015). As HIV is characterized by progressive cognitive and attentional deficits (Dawes et al., 2008; Heaton et al., 2010; Mothobi & Brew, 2012; Woods et al., 2009), and has recently been linked to diminished error-related suppression in some regions of the DMN (Flannery, Riedel, Salo, Poudel, et al., 2021), we hypothesized that HIV may be similarly associated with a SN-DMN bias and that this bias may have implications for certain cognitive functions. Further, recent work has demonstrated interacting HIV and CB use effects on both task-based activity (Meade et al., 2019) and resting-state functional connectivity (rsFC) of a major hub of the SN, the insula (Flannery, Riedel, Salo, Hill-Bowen, et al., 2021), and abnormal functioning in regions comprising the CEN has been linked to executive functioning deficits among PLWH (Castelo et al., 2006; Ernst et al., 2003; Ipser et al., 2015). If the SN biases neurocognitive resources toward internal processes associated with DMN function, some salient task-related events may go undetected. Indeed, evidence indicates that reduced RAIs correlate with diminished task performance *and* reduced task-induced suppression of DMN regions suggesting that alterations in rsFC, as indexed by the RAI, are linked with alterations in task-based performance and brain activity (Lerman et al., 2014).

In line with the triple network model (B. Menon, 2019; V. Menon, 2011), accumulating neuroimaging evidence links neuropsychiatric conditions not only with altered network-level rsFC (Alexopoulos et al., 2012; Bartova et al., 2015; Bednarski et al., 2011; Bonavita et al., 2017; Chang et al., 2014; Gauffin et al., 2013; Høgestøl et al., 2019; Lee et al., 2017; Liddle et al., 2011; Liu et al., 2018; Oyegbile et al., 2019; Peterson et al., 2009; Schilbach et al., 2016; Sutherland et al., 2012; Verfaillie et al., 2018; K. Wang et al., 2019; Y. Wang et al., 2013; Whitfield-Gabrieli & Ford, 2012; Wu et al., 2011; Yin et al., 2016; R. Zhang & Volkow, 2019; Zhou et al., 2016), but also with altered task-based network activity (Bartova et al., 2015; Bednarski et al., 2011; Liddle et al., 2011; Peterson et al., 2009). While small, albeit consistent, changes in brain network interactions distinguish task from resting states, task-evoked activity is closely related to resting-state organization (Cole et al., 2014; Smith et al., 2009), suggesting an intrinsic architecture of functional brain organization. As such, examining both resting-state network interactions and task-induced network activity may facilitate a more complete understanding of neurobiological contributors to cognitive alterations among neuropsychiatric conditions. In particular, lack of task-induced DMN suppression may represent a common endophenotype across various conditions (Bartova et al., 2015; Bednarski et al., 2011; Gauffin et al., 2013; Liddle et al., 2011; Oyegbile et al., 2019; Peterson et al., 2009; Sutherland et al., 2012; Whitfield-Gabrieli & Ford, 2012; R. Zhang & Volkow, 2019; Zhou et al., 2016); however, altered DMN suppression has rarely been examined among PLWH (Flannery, Riedel, Salo, Poudel, et al., 2021). As DMN suppression may facilitate task attention (Hinds et al., 2013) and detection of salient stimuli (Singh & Fawcett, 2008), insufficient suppression likely contributes to errors and/or a lack of error recognition. Proper error recognition is vital for everyday functioning, as it facilitates behavioral adaptation to minimize future negative outcomes. Indicative of metacognitive difficulties, PLWH often underreport their cognitive failures when compared with objective behavioral measures (Bassel et al., 2002; Hinkin et al., 1996; Van Gorp et al., 1991; Vance et al., 2008), suggesting compromised error recognition. Additionally, CB use may exacerbate error processing deficits among PLWH, as evidence has also demonstrated diminished error awareness among chronic CB users (Hester et al., 2009). Given that poor error awareness may impact disease management (e.g., taking medications) and everyday functioning, we sought to clarify the interrelations between network-level rsFC interactions, error awareness, and error-related brain activity among PLWH.

## METHODS

### Summary

We first examined the independent and combined effects of HIV and CB on rsFC utilizing the RAI as a measure of relative SN-DMN and SN-CEN interactions. Second, to delineate the behavioral implications linked with altered rsFC, we examined relations between rsFC and a task-based behavioral measure of error awareness. Third, to understand the relation between resting-state and task-based network functioning, we examined relations between rsFC and error-related brain activity during a Go/NoGo task variant. Regarding group effects, we expected to observe reduced RAI values among PLWH and CB users when compared with controls indicative of altered rsFC. Regarding behavioral and brain activity implications, we expected rsFC alterations to be linked with decreased error awareness and correlate with error-related brain activity.

### Participants

A sample of 106 participants was stratified into four groups based on HIV serostatus and CB use history (co-occurring: HIV+/CB+, *n* = 32; HIV-only: HIV+/CB−, *n* = 28; CB-only: HIV−/CB+, *n* = 24; controls: HIV−/CB−, *n* = 22). Demographic, descriptive, and fMRI data from this sample are also reported elsewhere (Flannery, Riedel, Salo, Hill-Bowen, et al., 2021; Flannery, Riedel, Salo, Poudel, et al., 2021). Participants were recruited from community-based organizations providing health-care services throughout Miami-Dade County. Participants were 18–60 years old to minimize the presence of other chronic conditions (e.g., hypertension, diabetes), as well as the potential interactive effect of HIV and aging on neurocognition (Morgan et al., 2012; Saloner et al., 2019; Seider et al., 2016; Valcour et al., 2011; Wendelken & Valcour, 2012). Additional exclusionary criteria included the following: current hepatitis C infection, English nonfluency or illiteracy, less than an eighth-grade education level, severe learning disability, significant neurological conditions (e.g., cerebrovascular issues, brain tumor, brain lymphoma, seizures, multiple sclerosis), severe head trauma with loss of consciousness >30 min, severe mental illness with psychotic or paranoid symptoms, or MRI contraindications.

PLWH in this study were diagnosed with HIV 9.3 ± 8.9 (mean ± *SD*) years prior to assessment and had no history of opportunistic infections affecting the central nervous system, and the majority (94.3%) were taking antiretroviral medications. All CB-using participants reported a history of regular use (operationalized as at least once per week for three straight months) and used at least 20 times in the past year. CB-non-using participants met the following criteria: no history of CB dependency, no CB use in the past month, and a negative urine THC screen. Past use of and dependence on other substances, including alcohol, nicotine, cocaine, amphetamines, benzodiazepines, or opioids was permitted across groups to provide a more representative and generalizable sample. However, participants were excluded if meeting criteria for current substance dependence (except CB and nicotine) as assessed via the substance use module of the *Diagnostic and Statistical Manual of Mental Disorders (DSM)-5* Structured Clinical Interview (First, 2014).

### Procedures

Study procedures were reviewed and approved by the Institutional Review Board of Florida International University. Following informed consent, we collected blood, behavioral, self-report, and MRI data across two study visits on different days. Participants were instructed to refrain from any substance use (including CB use among CB+ participants) for 24 hr before study visits to minimize acute pharmacological effects. Upon arrival at both visits, participants completed substance use screening including urine toxicology (Drug Check Cup, NXStep) and breathalyzer testing (AlcoMate Premium Breathalyzer). During the first visit, blood specimens were collected, and participants completed a battery of behavioral tests and self-report questionnaires. Among PLWH, blood samples were used to quantify HIV disease severity (HIV-1 viral load), immune function (CD4^+^ T-cell count, lymphocyte subset counts, total white blood cell count). The second visit occurred less than one month after the first and participants completed a 1-hr MRI scanning session and additional self-reports. Participants were compensated at the end of each visit.

### MRI Data Acquisition

MRI data were collected on a GE Healthcare Signa MR750, 3-Tesla scanner with 32-channel head coil. T1-weighted structural images were obtained using a magnetization-prepared rapid gradient-echo (MPRAGE) sequence (repetition time [TR] = 2,500 ms; echo time [TE] = 3.7 ms; flip angle [FA] = 12°; voxel size = 1 mm^3^). An 8-min resting-state scan with eyes closed was collected with 42 slices (3.4 mm thick) obtained in the axial plane using a T2*-weighted, single-shot, gradient-echo, echo-planar imaging (EPI) sequence sensitive to blood oxygen level–dependent (BOLD) effects (245 volumes, TR = 2,000 ms, TE = 30 ms, FA = 75°, field of view = 220 × 220 mm, 64 × 64 matrix, voxel size = 3.44 × 3.44 × 3.40 mm). These same EPI parameters were also used to collect six functional runs (169 volumes/run) while participants completed a Go/NoGo motor inhibition paradigm called the error awareness task (EAT; Hester et al., 2005; Hester et al., 2012; Hester et al., 2009; Hester et al., 2007). In the EAT, participants committed NoGo-errors (i.e., incorrectly pressed a button following a NoGo cue) of which they were either aware or unaware. Participants subsequently indicated error awareness by pressing an error signaling button on the trial following the error. The EAT allows for assessment of distinct brain activity linked with cognitive failures (i.e., NoGo-errors) and explicit error awareness. To achieve enough successful and unsuccessful NoGo trials for a sufficiently powered study, task difficulty was individually and dynamically adapted to maintain participants’ average NoGo-error rate between 45% and 50%. Participants performed this task during the four runs preceding and two runs following the resting-state scan. While EAT-associated brain activity has been reported elsewhere (Flannery, Riedel, Salo, Poudel, et al., 2021), here we focused on the EAT’s behavioral measure of error awareness and examined network responsivity to task errors.

### MRI Data Processing

Resting-state functional MRI (rs-fMRI) data were first denoised using dwidenoise (MRtrix3; Adhikari et al., 2018; Cordero-Grande et al., 2019; Tournier et al., 2019; Veraart, Fieremans, et al., 2016; Veraart, Novikov, et al., 2016), which utilizes Marchenko-Pastur principal component analysis (MPPCA) to estimate and remove Gaussian thermal noise from MRI data, including fMRI data (Adhikari et al., 2019), based on random matrix theory. The data were organized in BIDS format and additional preprocessing was performed with FMRIPrep v1.5.0 (Esteban et al., 2018), a Nipype-based tool (Gorgolewski et al., 2011) often employing Nilearn (Abraham et al., 2014). T1-weighted structural volumes were corrected for intensity nonuniformity (N4BiasFieldCorrection v2.1.0; Tustison et al., 2010) and skull-stripped (antsBrainExtraction.sh v2.1.0). Nonlinear registration (ANTs v2.1.0) was performed to spatially normalize T1-weighted volumes to the ICBM-152 asymmetrical template v2009c (Fonov et al., 2009). Functional data were slice-time corrected to the middle of each TR using 3dTshift (AFNI v16.2.07; Cox, 1996) and motion corrected using MCFLIRT (FSL v5.0.9; Jenkinson et al., 2002). Boundary-based registration (bbregister, FreeSurfer v6.0.1) was used to coregister functional images to corresponding T1-weighted volumes (2 × 2 × 2 mm isotropic voxels; Greve & Fischl, 2009) with 9 degrees of freedom. Lanczos interpolation (antsApplyTransforms ANTs v2.1.0) concatenated all motion-correction transformations (functional-to-anatomical, anatomical-to-template) and applied them in a single step. Physiological noise regressors were calculated applying aCompCor (Behzadi et al., 2007). Specifically, cerebral spinal fluid (CSF) and white matter (WM) masks were calculated in T1w space, within a mask excluding signal with cortical origin. Three aCompCor principal components were then calculated for both the CSF and the WM masks. Frame-wise displacement (FD; Power et al., 2013) was also calculated for each functional run using the Nipype implementation.

3dTproject (AFNI) was used to perform simultaneous nuisance regression and bandpass filtering. Nuisance regressors included the six aCompCor components (3 CSF, 3 WM; Muschelli et al., 2014), the six motion parameters, their derivatives, and TRs acquired during MRI stabilization (non-steady state) as determined by FMRIPrep. A 0.01 to 0.1 Hz bandpass filter was applied and TRs with FD greater than 0.35 mm were censored along with time points immediately preceding and following. Not all participants could be further processed because of temporal degrees of freedom violations in the denoising procedure (caused by nuisance regressors outnumbering observations), resulting in 93 remaining participants (co-occurring: HIV+/CB+, *n* = 28; HIV-only: HIV+/CB−, *n* = 27; CB-only: HIV−/CB+, *n* = 21; controls: HIV−/CB−, *n* = 17). An average of 6.2 ± 6.0% of volumes were excluded from each participant’s resting-state scan (Table S1 in the Supporting Information). Groups did not significantly differ in the number of censored volumes (HIV: *F*[1, 92] = 0.7, *p* = 0.4; CB: *F*[1, 92] = 1.0, *p* = 0.3; HIV × CB: *F*[1, 92] = 0.1, *p* = 0.8) or in mean FD (HIV: *F*[1, 92] = 0.04, *p* = 0.9; CB: *F*[1, 92] = 0.81, *p* = 0.4; HIV × CB: *F*[1, 92] = 0.001, *p* = 0.98). As motion is known to influence functional connectivity measures (Burgess et al., 2016; Power et al., 2012), mean FD was included as a covariate in group-level rsFC assessments.

Time series were then standardized (shifted to a zero mean and scaled to a unit variance) and averaged across voxels within four separate network masks (Nilearn, NiftiLabelsMasker.fit_transform). These network masks were defined with the Functional Imaging in Neuropsychiatric Disorders (FIND) atlas, which includes masks for an anterior salience network (SN), a dorsal default mode network (DMN), a left executive control network (labeled here as the central executive network; L.CEN), and a right executive control network (R.CEN) (Altmann et al., 2015; Shirer et al., 2012). Networks in this functional atlas were identified by applying independent component analysis (ICA; MELODIC, FSL) to resting-state data and visually identifying 14 canonical intrinsic functional connectivity networks based on prior work out of the 30 generated; the creation of this atlas is described in detail elsewhere (Shirer et al., 2012). While we did not have specific hypotheses regarding CEN laterality, we calculated separate RAIs for the left and right hemisphere consistent with prior work (Lerman et al., 2014; Reese et al., 2019; J. T. Zhang et al., 2017). We note that the RAI metric in these prior studies was calculated using network masks derived via an ICA-based (as opposed to an atlas-based) approach (Lerman et al., 2014; Moradi et al., 2020). As such, we also conducted ancillary analyses using an ICA-based approach to define the network masks of interest for calculating the RAI metrics (see Supplemental Text and Figures S1–S2 in the Supporting Information for methodological details and results).

Correlation coefficients between the four networks’ average time series were computed for each participant (Nilearn, ConnectivityMeasure.fit_transform) and used as a measure of functional coupling between networks. We then computed RAI values to quantify network interactions (Lerman et al., 2014) based on the hypothesized role of the SN toggling resources between the CEN and DMN. Specifically, the RAI metric integrates a positive SN-CEN correlation and a negative SN-DMN correlation such that higher RAI values indicate either positive synchronization of SN with CEN and/or negative synchronization of SN with DMN (Lerman et al., 2014). As done previously (Lerman et al., 2014), we calculated the RAI by first applying Fisher’s transform to correlation coefficients (*CC*) between the SN and the R.CEN (*CC*^*SN*−*R*. *ECN*^), L.CEN (*CC*^*SN*−*L*. *ECN*^), and DMN (*CC*^*SN*−*DMN*^) using **formula 1**. We then computed the RAI for the left and right CEN using **formula 2** and **formula 3**, respectively.

*Formula 1*.

$$f\left(CC\right)=0.5\times \ln\left(\frac{\left(1+CC\right)}{\left(1-CC\right)}\right)$$

*Formula 2*.

$$L.RAI=f\left({CC}^{SN-L.CEN}\right)-f\left({CC}^{SN-DMN}\right)$$

*Formula 3*.

$$R.RAI=f\left({CC}^{SN-R.CEN}\right)-f\left({CC}^{SN-DMN}\right)$$

### Network-Level rsFC: Group Effects

To assess HIV and CB main and interactive effects on RAI values, we performed HIV × CB general linear models (GLMs) including age, sex, mean FD, and whether the participant was a current cigarette smoker (NIC status; defined as smoking at least eight times in the last month) as covariates. We then performed follow-up analyses examining whether group differences in RAI values were driven by altered SN-CEN and/or SN-DMN rsFC. Specifically, we performed HIV × CB GLMs on participants’ standardized SN-L.CEN, SN-R.CEN, and SN-DMN correlation coefficients (*z*-scores) while controlling for the above covariates.

### Network-Level rsFC: Relation With Error Awareness Behavior

To link rsFC and behavior measures, we then considered relations between RAI values, SN-CEN rsFC, SN-DMN rsFC, and a behavioral measure of explicit error awareness from the EAT. Participants who did not meet the task performance criterion (>50% Go-errors) were excluded, resulting in a sample of 103 participants that had viable task data and a sample of 86 participants with both viable task-based and resting-state fMRI data. We first assessed group effects on error awareness (i.e., frequency of unaware errors) by performing an HIV × CB GLM with age, sex, and NIC status as covariates. One error awareness outlier was removed from all analyses including this variable. As the error awareness variable was positively skewed and included zero values, it was log_10_ transformed and a constant was added (log_10_[*x* + 1]) for this analysis. We then considered relations between RAI values, network-level (SN-CEN, SN-DMN) rsFC strength, and error awareness (*n* = 92). As the frequency of unaware errors variable also did not meet assumptions required for linear regression (Atkins & Gallop, 2007), we employed a negative binomial model (R, v.4.0.2) including age, sex, mean FD, and NIC status as covariates. A negative binomial model was selected over a zero-inflated or Poisson model as the test of dispersion indicated overdispersion (*p* < 0.00013) (Atkins & Gallop, 2007).

### Network-Level rsFC: Relation With Error-Related Brain Activity

To link resting-state and task-related brain activity, we then considered the relations between SN-CEN or SN-DMN rsFC and error-related activity during the EAT. First, we assessed whether network-level responsivity to EAT errors demonstrated HIV × CB effects and/or relationships with error awareness (paralleling the rsFC analyses above). To do so, the six EAT runs were preprocessed with FMRIPrep (reported in more detail elsewhere; see Flannery, Riedel, Salo, Hill-Bowen, et al., 2021). Time series were scaled to the voxel-wise mean (3dcalc), thereby allowing regression (*β*) coefficients to be interpreted as an approximation of percentage BOLD signal change (% BOLD Δ; Chen et al., 2017) from the implicit baseline. Data were entered into subject-level GLMs (3dDeconvolve, 3dREMLfit) that concatenated runs and modeled nuisance regressors (i.e., six motion-correction parameters and fourth-order polynomials capturing residual head motion and baseline trends in the BOLD signal) and three task-related regressors (NoGo-correct, NoGo-error, and Go-error) as impulse functions time-locked to stimulus onset and convolved with a hemodynamic response (gamma) function. As such, activity associated with task events represents activity over and above that of the ongoing Go trial period, similar to prior work (Hester et al., 2005; Hester et al., 2012; Hester et al., 2009). Average *β* coefficients associated with NoGo-error task events were extracted for each participant by averaging across all nonzero voxels within the four network masks.

To assess group effects on the error-related responsivity, we conducted HIV × CB GLMs on averaged NoGo-error *β* coefficients within each network of interest (DMN, SN, R.CEN, L.CEN), among participants with viable task data (*n* = 103). Age, sex, and NIC status were included as covariates. Second, we considered whether error-related network activity correlated with error awareness by again employing a negative binomial model among all participants with both viable task and rest data (*n* = 85, one outlier was removed), while controlling for the same covariates listed above. Finally, to directly link rsFC and task-related brain activity, we conducted partial Pearson’s correlations between rsFC values and error-related network activity controlling for mean FD during rest.

## RESULTS

### Group Characteristics

Demographic and drug use characteristics of this sample have been reported elsewhere (Flannery, Riedel, Salo, Hill-Bowen, et al., 2021), and are summarized below. Groups did not differ in terms of age, education, race, ethnicity (Table 1; *p* > 0.3), or history of major depressive episodes (Table S2 in the Supporting Information; *p* > 0.3). However, the HIV+ groups included a higher percentage of self-reported males (80% male) than did the HIV− groups (55.3% male; *p* = 0.006), consistent with national estimates regarding the sex distribution (81% male) of new HIV diagnoses (Centers for Disease Control and Prevention, 2020). This difference was driven by the female/male composition among the CB+ groups (HIV+/CB+ vs. HIV−/CB+: *χ*^2^[1, 92] = 6.6, *p* = 0.014), but not the CB− groups (HIV+/CB− vs. HIV−/CB−: *χ*^2^[1, 92] = 3.3, *p* = 0.1). Self-reported sex was included as a covariate in all group-level analyses. Of the PLWH in the study (*n* = 54), 64.8% had an undetectable viral load (i.e., <50 mRNA viral copies/mL) and 20.4% were classified with mild to moderate cognitive impairment, based on a validated neurocognitive screening index described in prior work (Carey et al., 2004). Groups composed of PLWH (i.e., HIV+/CB− vs. HIV+/CB+) did not differ when considering HIV-status measures (i.e., time since diagnosis, *n* with detectable viral load, *n* with AIDS diagnosis, viral load [copies of HIV-1 RNA/mL], *n* with mild to moderate cognitive impairment) or immune function (i.e., CD4^+^ T-cell count [cells/uL], CD8^+^ T-cell count, CD4^+^/CD8^+^ cell ratio, total T-lymphocytes, total white blood cell count; Table 1). CB-using groups were matched on self-reported measures of CB exposure (Table 1; *p* > 0.1), and groups were largely matched on other drug use characteristics including past dependence (Tables S3–S5 in the Supporting Information); however, CB-using groups reported more past month nicotine use (*F*[1, 92] = 9.7, *p* = 0.002). Thus, whether a participant was a current cigarette smoker (NIC) was included as a covariate in all group-level analyses.

**Table 1.** Participant demographic, cannabis, HIV disease characteristics, and other drug use. Data are expressed as either mean (standard deviation) or frequency across all participants or within specific groups. Drug use is the self-reported number of times using each drug in the given timeframe (i.e., past month, lifetime). HIV-1 viral load was assessed via the Abbott *RealTime* HIV-1 assay. Group effects were assessed via either an HIV × CB ANOVA or, for categorical variables, via chi-square tests (one comparing HIV+ vs. HIV− groups and one comparing CB+ vs. CB− groups). AA: African American, C: Caucasian, A: Asian, >1: more than one race. ^☨^Independent sample *t* test between CB+ groups. ^δ^All group effects in section assessed with independent sample *t* test between HIV+ groups, or for categorical variables, a chi-square test. ^ξ^Estimator of general cognitive impairment: *t* score < 40 for both: total recall on the Hopkins Verbal Learning Test–Revised (HVLT-R; Benedict et al., 1998; Brandt, 1991) and symbol search scores on the Wechsler Adult Intelligence Scale–Fourth edition (WAIS-IV; Wechsler, 2008), or *t* score < 35 for either test (Carey et al., 2004).

	**All participants**	**HIV+/CB+**	**HIV+/CB−**	**HIV−/CB+**	**HIV−/CB−**	**Group effects (*p*)**		
	*n* = 93	*n* = 28	*n* = 26	*n* = 22	*n* = 17	HIV × CB	HIV	CB
**Demographic**								
Age	34.8 (10.3)	33.3 (7.4)	37.5 (13.2)	32.8 (10.1)	35.8 (9.3)	0.7	0.9	0.2
Education (years)	13.8 (2.3)	13.8 (2.0)	13.6 (2.8)	13.8 (2.7)	14.4 (1.2)	0.4	0.4	0.7
Male, female	65, 28	26, 2	18, 8	14, 8	7, 10	–	**0.006**	**0.04**
AA, C, >1	48, 41, 4	14, 14, 0	14, 10, 2	11, 10, 1	9, 7, 1	–	0.5	0.3
Hispanic/Latinx	35	10	9	8	6	–	0.8	0.6
**Cannabis use**								
Age regular use	19.6 (6.7)	21.1 (6.5)	–	18.2 (7.1)	–	–	0.1^☨^	–
Years regular use	14.8 (11.5)	12.1 (8.9)	–	15.5 (12.1)	–	–	0.3^☨^	–
Past month (times)	12.8 (14.0)	23.4 (9.8)	0	24.4 (10.1)	0		0.5^☨^	
Lifetime (times)	2,141 (3,112)	3,556 (2,969)	282 (1,024)	4,182 (3,852)	7.8 (23)	0.4	0.7	**<0.001**
**HIV disease characteristics** ^δ^								
Years since HIV diagnosis	9.3 (8.9)	7.8 (6.9)	10.8 (10.6)	–	–	–	–	0.2
*n* with undetectable viral load (<50 copies/mL)	35	17	18	–	–	–	–	0.5
*n* with AIDS diagnosis	6	4	2	–	–	–	–	0.7
Viral load (copies of HIV RNA/mL)	14,691.9 (57,211.4)	18,254.8 (69,658.7)	10,854.9 (40,853.7)	–	–	–	–	0.6
*n* with mild to moderate cognitive impairment^ξ^	11	5	6	–	–	–	–	0.6
**Immune function** ^δ^								
CD4^+^ T-cell count (cells/uL)	659.2 (305.9)	672.1 (282.3)	645.4 (334.6)	–	–	–	–	0.8
CD8^+^ T-cell count (cells/uL)	1,064.9 (562.4)	993.2 (445.9)	1,142.2 (666.2)	–	–	–	–	0.3
CD4^+^/CD8^+^ cell ratio	0.755 (0.1)	0.774 (0.1)	0.736 (0.1)	–	–	–	–	0.8
Total T-lymphocytes (cells/uL)	1,786.1 (676.9)	1,728.9 (546.5)	1,847.7 (800.7)	–	–	–	–	0.5
Total white blood cells (thousand cells/uL)	6,533.3 (2,002.0)	6,167.9 (1,962.1)	6,926.9 (2,007.1)	–	–	–	–	0.2
**Other drug use (past month, times)**								
Alcohol	2.2 (3.3)	2.2 (2.7)	2.2 (4.2)	2.9 (3.2)	1.4 (2.9)	0.3	0.9	0.3
Cocaine	0.02 (0.1)	0.1 (0.3)	0	0	0	0.2	0.2	0.2
Nicotine	4.3 (10.2)	7.2 (12.7)	1.5 (6.0)	7.2 (12.8)	0	0.7	0.7	**0.002**
Current cigarette smoker (count)	16	8	3	5	0	–	0.9	0.08

### Network-Level rsFC: Group Effects

**Figure 1.**
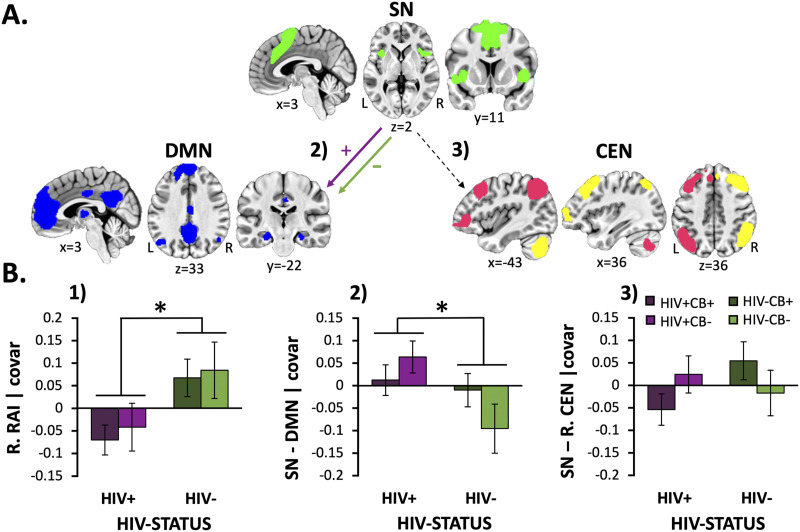
HIV-associated effects on network-level resource allocation index and rsFC (SN-DMN and SN-CEN) metrics. (A) Schematic of network-level resting-state functional connectivity (rsFC) differences between HIV+ and HIV− participants. People living with HIV (PLWH) showed *increased* rsFC (purple [+] arrow) between the salience network (SN; green) and the default mode network (DMN; blue) relative to that from HIV− participants (green [−] arrow). The gray dashed arrow indicates nonsignificant group differences when considering rsFC between the SN and central executive network (CEN; pink = L.CEN, yellow = R.CEN). (B.1) PLWH (vs. HIV− participants) showed reduced resource allocation index (RAI) values when considering both the right (*p* = 0.008) and left hemisphere (data not displayed, *F*[1, 92] = 4.3, *p* = 0.042). (B.2) HIV-associated RAI reductions were driven by *increased* SN-DMN rsFC among PLWH (vs. HIV− participants; *p* = 0.027). (B.3) On the other hand, significant group differences were not detected when considering SN-R.CEN or SN-L.CEN (data not displayed) rsFC values (*p* > 0.3), which were the second aspect contributing to the composite RAI value. Unstandardized residuals are plotted after regressing effects of age, sex, mean FD, and NIC status. Error bars = standard error of the mean.

A main effect of HIV was observed when considering the RAI metrics from both the right (*F*[1, 92] = 7.5, *p* = 0.008, Figure 1A) and the left hemispheres (F[1, 92] = 4.3, *p* = 0.042, data not shown). Specifically, PLWH displayed reduced RAI when controlling for CB group membership, age, sex, mean FD, and NIC status (Figure 1B.1). This HIV-associated RAI reduction did not appear to differ among CB users and nonusers (*HIV* × *CB interaction*: right, *F*[1, 92] = 0.01, *p* = 0.9; left, *F*[1, 92] = 0.2, *p* = 0.7; *CB main effect*: right, *F*[1, 92] = 0.3, *p* = 0.6; left, *F*[1, 92] = 0.4, *p* = 0.5). Follow-up analyses indicated that this HIV-associated RAI reduction was driven by *increased* SN-DMN rsFC among PLWH (vs. HIV− participants; Figure 1B.2; *F*[1, 92] = 5.0, *p* = 0.027). While the HIV × CB interaction (*F*[1, 92] = 2.9, *p* = 0.09) and CB main effects (*F*[1, 92] = 0.2, *p* = 0.6) did not reach significance, visual inspection of SN-DMN rsFC values across groups and exploratory follow-up *t* tests within each CB group suggested that HIV-related group differences were most pronounced among CB *nonusers* (HIV+/CB− vs. HIV−/CB−: *t*[41] = −2.5, *p* = 0.016) and appeared attenuated among CB *users* (HIV+/CB+ vs. HIV−/CB+: *t*[48] = −0.2, *p* = 0.8). No significant group differences were detected when considering right (Figure 1B.3) or left SN-CEN rsFC (data not shown) (*p* > 0.3). We also performed follow-up exploratory GLMs, among HIV+ participants (*n* = 54), assessing relationships between RAI values, rsFC, and HIV disease characteristics (viral load, duration since diagnosis, CD4^+^ T-cell count, CD8^+^ T-cell count, total T-lymphocytes, total white blood cell count), when controlling for CB group membership, age, sex, mean FD, and NIC status. We did not observe any significant relationships between network rsFC measures and HIV disease characteristics (*p* > 0.07).

Overall, similar outcomes and interpretations were obtained when employing an ICA-based approach to define the four network masks of interest and reassessing rsFC metrics across groups; however, PLWH displayed significantly reduced rsFC between the SN and L.CEN compared with HIV− participants (*F*[1, 92] = 4.2, *p* = 0.04), while this same effect was not observed using the atlas-based approach (Figures S1 and S2 in the Supporting Information). As this inconsistency may be due to the relatively more bilateral nature of the ICA-based L.CEN network mask when compared with the atlas-based L.CEN mask, we performed exploratory follow-up analyses assessing group differences in SN-CEN rsFC when merging both atlas-based CEN masks to create a bilateral CEN mask. However, we did not find any significant group effects on SN-CEN rsFC when utilizing this bilateral CEN mask (*p* > 0.1).

### Network-Level rsFC: Relation With Error Awareness

**Figure 2.**
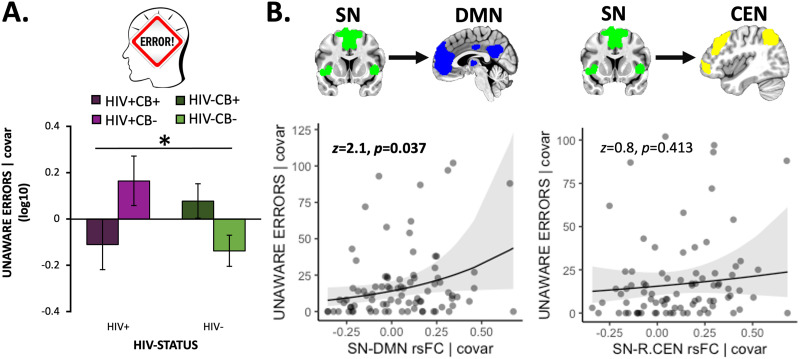
Error awareness behavior was linked with SN-DMN rsFC strength. (A) We observed an HIV × CB interaction (*F*[1, 101] = 5.9, *p* = 0.017) when considering error awareness (operationalized as the log-transformed number of unaware errors [log_10_(*x* + 1)]) such that both the HIV-only (HIV+/CB−) and CB-only (HIV−/CB+) groups (relative to controls, HIV−/CB−) failed to indicate awareness of more errors, whereas the co-occurring (HIV+/CB+) group displayed fewer unaware errors more akin to that of controls. (B) Increased salience network–default mode network (SN-DMN) resting-state functional connectivity (rsFC) correlated with more unaware errors in the Go/NoGo task (*n* = 85, *b* = 1.7 [0.8], *z* = 2.1, *p* = 0.037). In contrast, SN rsFC with the left central executive network (L.CEN, data not shown: *b* = 0.6 [0.7], *z* = 0.8, *p* = 0.430) or right CEN (*b* = 0.6 [0.8], *z* = 0.8, *p* = 0.413) was not linked with error awareness. Unstandardized residuals are plotted after regressing out effects of covariates. Error bars = standard error of the mean.

When considering a behavioral measure of error awareness during the modified Go/NoGo task, we observed a significant HIV × CB interaction (*F*[1, 101] = 5.9, *p* = 0.017, *η*_*p*_^2^ = 0.059) when controlling for covariates (Figure 2A). Specifically, visual inspection and follow-up *t* tests indicated that both the HIV-only (*p* = 0.041; HIV+/CB−) and CB-only (*p* = 0.020; HIV−/CB+) groups committed more unaware errors, compared with controls (HIV−/CB−), whereas the co-occurring (HIV+/CB+) group had fewer unaware errors more similar to that of controls (*p* = 0.767). Importantly, negative binomial models examining relations between network-level rsFC and error awareness indicated that higher SN-DMN rsFC was linked with more unaware errors (i.e., reduced error awareness; Figure 2B; *b* = 1.7 [0.8], *z* = 2.1, *p* = 0.037). Neither right RAI, left RAI (Figure S3A in the Supporting Information) nor SN-CEN rsFC (Figure 2B) displayed a significant relationship with error awareness (*p* > 0.4). These results did not significantly change when including HIV and CB group membership as covariates. These outcomes suggest that more SN-DMN rsFC was linked with consequences for task performance, namely reduced awareness of commission errors. Given the severe non-normality of the unaware error variable, we believe that the negative binomial model is the proper model for this analysis; however, to increase transparency, we also reran this analysis employing a general linear regression while controlling for the same covariates (Figure S3B in the Supporting Information). The relationship between SN-DMN rsFC and unaware errors no longer reached significance (*p* = 0.082). We also performed follow-up exploratory analyses, among HIV+ participants, assessing relationships between error awareness and HIV disease characteristics when controlling for CB group membership, age, sex, mean FD, and NIC status; however, no significant relationships were observed (*p* > 0.05).

### Network-Level rsFC: Relation With Error-Related Brain Activity

**Figure 3.**
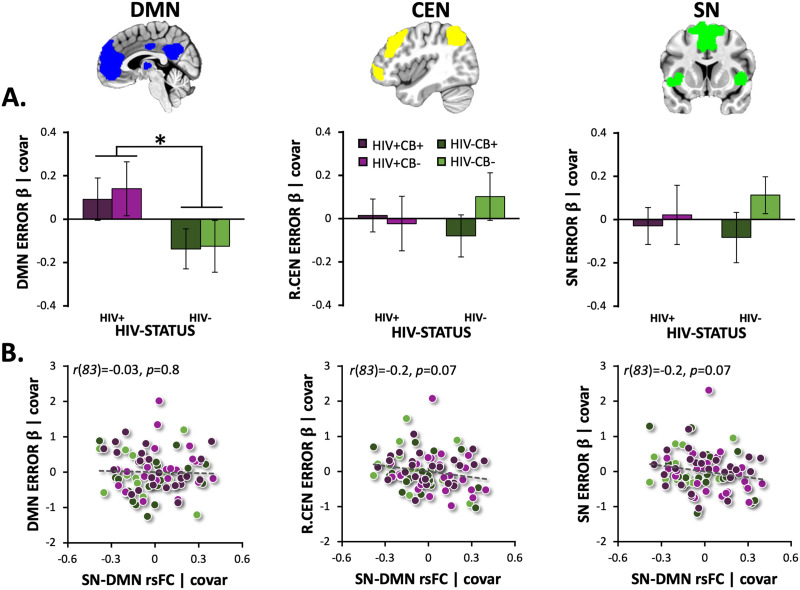
HIV-associated alterations in error-related DMN suppression and relationships with SN-DMN rsFC strength. (A) People living with HIV (PLWH) had significantly higher error-related default mode network (DMN) activity (i.e., reduced DMN suppression; average NoGo-error *β* coefficients) than did HIV− participants (*F*[1, 102] = 5.1, *p* = 0.026; *n* = 103). Error-related right central executive network (R.CEN), left CEN (L.CEN, data not displayed), and salience network (SN) activity did not differ between HIV groups (*p* > 0.3). (B) Resting-state functional connectivity (rsFC) between the SN and DMN did not correlate with error-related DMN (*r*(83) = −0.03, *p* = 0.8), R.CEN (*r*(83) = −0.2, *p* = 0.07), L.CEN (*r*(83) = −0.1, *p* = 0.2; data not shown), or SN activity (*r*(83) = −0.2, *p* = 0.07). Unstandardized residuals are plotted after regressing out effects of covariates. Error bars = standard error of the mean.

When examining error-related *β* coefficients from the Go/NoGo task within each network of interest (DMN, SN, R.CEN, L.CEN), we observed a significant main effect of HIV for the DMN, such that PLWH (vs. HIV− participants) showed reduced DMN suppression (*F*[1, 102] = 5.1, *p* = 0.026; Figure 3A). This reduced error-related DMN suppression among PLWH was not impacted by CB use (HIV × CB: *F*[1, 102] = 0.3, *p* = 0.865; CB: *F*[1, 102] = 0.1, *p* = 0.739). Error-related DMN (*z* = −1.4, *p* = 0.167), R.CEN (*z* = −1.6, *p* = 0.112), L.CEN (data not shown: *z* = −1.4, *p* = 0.169), and SN (*z* = 0.2, *p* = 0.813) responsivity in the error awareness task (EAT) was not significantly associated with error awareness, while controlling for sex, age, and NIC status (Figure S4 in the Supporting Information). Finally, when controlling for mean FD (during rest), we did not detect any relations between rsFC (SN-DMN, SN-CEN) and error-related network activity (*p* > 0.8; Figure 3B), contrary to our hypothesis.

## DISCUSSION

We characterized large-scale brain network interaction patterns linked with HIV and CB use and examined implications for behavior and task-based brain function. We assessed HIV and CB-related effects on RAIs, a measure previously used to quantify interactions across three large-scale networks: the SN, CEN, and DMN. We observed bilaterally reduced RAIs among PLWH driven by *increased* SN-DMN rsFC, but not SN-CEN rsFC. No significant CB-related effects on RAI and rsFC measures were detected. Our findings link SN-DMN rsFC strength with an objectively measured behavioral metric; as such, rsFC was associated with error awareness during a Go/NoGo task variant. Specifically, increased SN-DMN coupling correlated with reduced error awareness (i.e., more unaware errors) across all participants. Contrary to our hypothesis, SN-DMN rsFC did not correlate with error-related DMN suppression. However, we observed that PLWH displayed reduced error-related DMN suppression compared with HIV− controls. These results demonstrate a pattern of dysregulated network function among PLWH and highlight implications for error awareness.

### Reduced RAI Among PLWH

The bilaterally reduced RAIs observed among PLWH are similar to those previously observed across substance use and neuropsychiatric disorders (Alexopoulos et al., 2012; Bartova et al., 2015; Bednarski et al., 2011; Bonavita et al., 2017; Chang et al., 2014; Gauffin et al., 2013; Høgestøl et al., 2019; Lee et al., 2017; Liddle et al., 2011; Liu et al., 2018; Oyegbile et al., 2019; Peterson et al., 2009; Schilbach et al., 2016; Sutherland et al., 2012; Verfaillie et al., 2018; K. Wang et al., 2019; Y. Wang et al., 2013; Whitfield-Gabrieli & Ford, 2012; Wu et al., 2011; Yin et al., 2016; R. Zhang & Volkow, 2019; Zhou et al., 2016). Nicotine and other drug-dependent individuals have displayed reduced RAIs during acute withdrawal (Lerman et al., 2014; Reese et al., 2019). However, Moradi et al.’s (2020) recent work questioned the RAI as a reliable biomarker for substance use disorders following null effects among stimulant and/or opiate users that had been abstinent for, in some cases, multiple months (mean of 108 days, ranging from 4 to 365 days). However, we note that prior examinations of RAI changes among dependent substance users suggest that they may be linked with certain cognitive symptoms of an acute withdrawal state (cognitive control among cigarette smokers [Lerman et al., 2014], craving among individuals with internet gaming disorder [J. T. Zhang et al., 2017], and distress tolerance among cocaine users [Reese et al., 2019]), and these symptoms were not considered in Moradi et al.’s (2020) study (Lerman et al., 2014; Reese et al., 2019; J. T. Zhang et al., 2017). Many of the cognitive deficits characterizing withdrawal are hypothesized to stem from an inability to suppress attention toward internal craving and aversive somatic withdrawal symptoms (Ashare et al., 2014; Lerman et al., 2014; Shoaib & Bizarro, 2005; Sutherland et al., 2012; Wesnes et al., 2013). Our results point toward a similar mechanism at play among PLWH that could account for certain cognitive deficits that persist in the post–antiretroviral therapy era. Specifically, deficits reported in learning, memory, and performance on cognitive tasks involving executive function may be manifestations of a more general attentional impairment characterized by an inability to suppress attention toward physical/emotional HIV symptomology and/or other task-irrelevant, intrusive thoughts linked with the DMN.

### Increased SN-DMN rsFC Among PLWH

This interpretation is further supported by our observation that HIV-associated RAI reductions were primarily driven by PLWH (vs. HIV− participants) presenting with higher SN-DMN synchrony. Prior work demonstrating altered network organization among PLWH (Abidin et al., 2018; Hall et al., 2021; Minosse et al., 2021) has also often highlighted alterations involving the DMN (Thomas et al., 2015; Zhuang et al., 2017). For example, one study found that treatment naïve PLWH displayed significantly reduced rsFC within the DMN when compared with controls (Zhuang et al., 2017), while another study utilizing graph theoretic metrics found alterations in closeness centrality (a metric indicating connectiveness with the rest of the brain) within the DMN and frontoparietal network among PLWH (Thomas et al., 2015). Our findings are also generally consistent with prior work among individuals living with other conditions leading to chronic inflammation in the central nervous system (i.e., multiple sclerosis [MS]). Specifically, people living with MS displayed increased rsFC between DMN regions and those comprising the SN, and such rsFC alterations correlated with symptom severity (Bonavita et al., 2017; Høgestøl et al., 2019). Altered DMN rsFC also has been consistently linked with Alzheimer’s disease (AD; Damoiseaux et al., 2012; Jones et al., 2016) and other neurodegenerative disorders (Alexopoulos et al., 2012; Liu et al., 2018; Yin et al., 2016). For example, emerging evidence suggests that mild cognitive declines that precede the onset of progressive deterioration in AD may be associated with an initial *increase* in DMN rsFC followed by subsequent *decreases* (Damoiseaux et al., 2012; Jones et al., 2016; Wu et al., 2011). Accordingly, changes in DMN rsFC may be predictive of future cognitive impairments before neuropsychological performance falls outside a normative range (Verfaillie et al., 2018; Y. Wang et al., 2013). Indeed, when considering individuals with a family history of AD, early subjective cognitive decline is associated with *increased rsFC* between the DMN and regions of the medial temporal lobe memory system (Verfaillie et al., 2018). In contrast, individuals with mild cognitive impairments and those reporting cognitive complaints (despite normal neuropsychological performance) appear to display *decreased rsFC* between the DMN and right hippocampus relative to age-matched controls (Y. Wang et al., 2013). In light of such AD findings, we suggest that future work could consider longitudinal changes in DMN rsFC across HIV disease phases with attention towards potential nonlinear associations with cognitive decline (Damoiseaux et al., 2012; Jones et al., 2016; Wu et al., 2011).

Despite prior reports of reduced RAI values associated with certain symptoms of addiction disorders, we did not observe significant CB effects on RAI values or SN-DMN rsFC. This null result, corresponding with Moradi et al.’s (2020) null findings, may be due to CB users in our sample not being in an acute withdrawal state, or interacting influences of HIV that we did not have the power to detect. Interestingly, while we did not detect significant HIV × CB interaction effects, visual inspection and exploratory follow-up *t* tests indicated that the CB-using HIV+ group displayed reduced SN-DMN rsFC, more similar to that of the control group, when compared with the non-using PLWH group. These exploratory observations may support prior work observing CB normalizing effects among PLWH when considering task-based activity within SN regions (Meade et al., 2019) and rsFC metrics centered on the insula (Flannery, Riedel, Salo, Hill-Bowen, et al., 2021). As identifying specific brain network interaction patterns linked with both CB use and possible CB normalizing effects among PLWH could help inform clinical practices regarding medicinal CB use among this population, future work should further examine aspects of CB use among PLWH and its effects on network rsFC.

### Network-Level rsFC: Relation With Error Awareness

While both the HIV-only (HIV+/CB−) and CB-only (HIV−/CB+) groups displayed diminished error awareness relative to controls, the co-occurring (HIV+/CB+) group’s error awareness was more similar to that of the controls. These outcomes align with prior observations of a partially normalizing effect of CB use on HIV-associated brain function alterations and error processing (Flannery, Riedel, Salo, Hill-Bowen, et al., 2021; Hall et al., 2021; Meade et al., 2019). As chronic inflammation in the central nervous system is one mechanism through which HIV may lead to progressive cognitive declines (Benatti et al., 2016; Boerwinkle & Ances, 2019), it has been hypothesized that the anti-inflammatory properties of CB could offer some benefits among PLWH (Burstein, 2015; Ellis et al., 2009; Gallily et al., 2018; Watson et al., 2020). Supporting this notion, CB use has been linked with reduced inflammatory biomarkers in cerebral spinal fluid and blood (Castro et al., 2019; Ellis et al., 2020; Rizzo et al., 2018), and PLWH frequently report using CB to relieve somatic complaints, and anxious or depressed moods (Harris et al., 2014; Towe et al., 2020). That said, continued research is still needed to better understand the impact of CB use on interacting physical, affective, and neurocognitive symptoms of HIV (Bonnet & Preuss, 2017; Bovasso, 2001; Okafor et al., 2019; Thames et al., 2016).

Additionally, we observed that higher SN-DMN rsFC was associated with diminished error awareness. These outcomes highlight the relevance of network interactions assessed at rest for error monitoring during tasks. While increased SN-DMN rsFC has not previously been linked to compromised error awareness, it has been linked to attentional problems in the context of ADHD, with medication down regulating DMN rsFC with regions comprising the SN (Biskup et al., 2016; Querne et al., 2017). Given the theorized role of the SN in toggling neurocognitive resources across large-scale brain networks, we hypothesized that SN-DMN rsFC has implications for task-induced DMN suppression. As task-induced DMN suppression is thought to support task vigilance and detection of salient stimuli (Singh & Fawcett, 2008), it is likely also vital for error recognition.

### Network-Level rsFC: Relation With Error-Related Brain Activity

While research has shown that properties of functional networks identified during rest reflect a similar functional network architecture during tasks, it remains unclear how the two are related (Cole et al., 2014). To more fully understand altered network organization among PLWH, we examined relations between rsFC and task event-induced network responsivity. We did not observe a relation between measures of network function during rest and those during task. While it is possible that both alterations in rsFC and task-based responsivity are associated with a certain condition, in this case, living with HIV, the two may be unrelated consequences. However, it should be noted that rest and task are distinct cognitive states and functional connectivity and task event responsivity are different quantifications of brain function with distinct data preparation and analysis techniques (Stevens, 2016). Thus, failure to detect a relation between specific alterations in both could be influenced by multiple differences in these two variables and their quantification. Further, while rest and task data were collected during the same scan session (1.5 hr), a lack of within-subject reliability/stability across scans has previously been documented (Pannunzi et al., 2017). Whether rsFC alterations, observed among certain populations, represent a broad-spectrum alteration that also impacts brain processes probed during tasks is a potentially important scientific question that could help advance understanding of neurobiological consequences of HIV. Future work should continue to consider how to best examine relations between the degree of regional responsivity to stimuli/events and functional synchrony between of two or more regions.

Reduced task-induced DMN suppression has been linked with various neurocognitive disorders (Gauffin et al., 2013; Oyegbile et al., 2019; Whitfield-Gabrieli & Ford, 2012), including addiction (Bednarski et al., 2011; Sutherland et al., 2012; R. Zhang & Volkow, 2019), ADHD (Liddle et al., 2011; Peterson et al., 2009), major depressive disorder (Bartova et al., 2015), and schizophrenia (Zhou et al., 2016). Additionally, our own recent work characterized reduced error-related suppression of the medial prefrontal cortex (mPFC) and posterior cingulate cortex (PCC; two primary nodes of the DMN) among PLWH (Flannery, Riedel, Salo, Poudel, et al., 2021). Here, we replicated these findings, when adopting a network-level (as opposed to a regional) analytic framework, such that PLWH demonstrated reduced error-related DMN suppression. A robust body of work indicates that task-based DMN suppression plays a critical role in monitoring task stimuli (Hinds et al., 2013) and is related to increasing cognitive demands (Allen et al., 2013; Leech et al., 2011) and task performance (Anticevic et al., 2012; Li et al., 2007). Given our finding that SN-DMN rsFC was both heightened among PLWH and associated with reduced error awareness, we expected that less error-related DMN suppression among PLWH would be similarly related to error awareness; however, we did not detect an association between DMN suppression and error awareness.

### Limitations

While we elected to utilize the RAI metric to quantify network-level interactions consistent with previous drug use–related studies (Lerman et al., 2014; Sutherland et al., 2012), this work should be considered in light of limitations. First, recent work by Moradi et al. (2020) highlighted the impact of mask definition on RAIs. To address mask definition–related variability, we used two different strategies to define our network masks (atlas-based and ICA-based) and came to similar conclusions. Second, we note that, while past use of and dependence on substances other than cannabis was permitted across each group to provide a more representative and generalizable sample, the CB-using groups reported significantly more past month nicotine use than non-cannabis-using groups. Additionally, because of the disproportionate HIV diagnoses among males in the United States, and challenges recruiting among this population, our subject groups did not have an equal distribution of self-reported males and females. We statistically controlled for both sex and current cigarette smoking status but were unable to rule out potentially confounding effects that may have still influenced results. Finally, while our work controlled for age effects, we did not have a large enough sample to assess complex interactive effects of age in the context of HIV and CB use; however, these interactive trajectories may be of interest in future research.

### Conclusions

Our results demonstrate heightened SN-DMN rsFC among PLWH (vs. HIV− participants) that was linked with diminished error awareness behavior across all participants, but contrary to our hypothesis, was not linked with error-related network responsivity. However, we did observe significantly reduced error-related DMN suppression among PLWH. Interestingly, our results also displayed a significant HIV × CB interaction on error awareness behavior such that both the HIV-only (HIV+/CB−) and CB-only (HIV−/CB+) groups exhibited diminished error awareness relative to controls, whereas the co-occurring (HIV+/CB+) group displayed error awareness more similar to that of the controls. These findings demonstrate a pattern of dysregulated network function among PLWH that has been associated with other neurocognitive conditions, further highlighting the importance and ubiquity of this functional network perspective. Our results also suggest that such dysregulation may exist during both resting state and task performance and reflect an inability to disengage irrelevant mental operations, ultimately hindering error awareness. As monitoring errors is vital for everyday functioning and critical disease management behaviors, we speculate that interventions facilitating DMN suppression (e.g., mindfulness-based practices, working memory training; Garrison et al., 2015; Salmi et al., 2018) may be beneficial for PLWH and the challenges they face.

## ACKNOWLEDGMENTS

We thank the FIU Instructional and Research Computing Center (IRCC, https://ircc.fiu.edu) for providing access to the HPC computing resources that contributed to the generation of the research results reported herein.

## DATA AND CODE AVAILABILITY

The authors have released all code associated with this manuscript. Code and tabular data are available on GitHub (https://github.com/Flanneryg3/HIVCB_ProjectCode; Flannery, 2021).

## SUPPORTING INFORMATION

Supporting information for this article is available at https://doi.org/10.1162/netn_a_00241.

## AUTHOR CONTRIBUTIONS

Jessica S. Flannery: Conceptualization; Formal analysis; Project administration; Writing – original draft. Michael C. Riedel: Formal analysis; Methodology. Lauren D. Hill-Bowen: Writing – review & editing. Ranjita Poudel: Project administration; Writing – review & editing. Katherine L. Bottenhorn: Writing – review & editing. Taylor Salo: Data curation; Formal analysis; Writing – review & editing. Angela R. Laird: Funding acquisition; Writing – review & editing. Raul Gonzalez: Funding acquisition; Project administration; Writing – review & editing. Matthew T. Sutherland: Conceptualization; Funding acquisition; Project administration; Supervision; Writing – review & editing.

## FUNDING INFORMATION

Matthew T. Sutherland, Foundation for the National Institutes of Health (https://dx.doi.org/10.13039/100000009), Award ID: K01DA037819. Raul Gonzalez, Foundation for the National Institutes of Health (https://dx.doi.org/10.13039/100000009), Award ID: R01DA033156. Matthew T. Sutherland, Foundation for the National Institutes of Health (https://dx.doi.org/10.13039/100000009), Award ID: U54MD012393. Matthew T. Sutherland, Foundation for the National Institutes of Health (https://dx.doi.org/10.13039/100000009), Award ID: R01DA041353. Angela R. Laird, National Science Foundation (https://dx.doi.org/10.13039/501100008982), Award ID: 1631325.

## TECHNICAL TERMS

Default mode network (DMN): Large-scale brain network most active during internally focused thought and deactivated when focused on external tasks.
Central executive network (CEN): Large-scale brain network most active during externally oriented, attentionally demanding cognitive tasks, generally anticorrelated with DMN activity.
Salience network (SN): Large-scale brain network thought to dynamically prioritize internally versus externally oriented cognition by toggling relative activity between the DMN and CEN, respectively.
Resource allocation index (RAI): Previously developed metric quantifying synchrony between the DMN and SN relative to that between the CEN and SN.
Default mode network (DMN) suppression: Commonly observed phenomena of reduced DMN activity when performing neuroimaging tasks, where greater activity decreases scale with increasing task difficulty.
Resting-state functional connectivity (rsFC): Quantification of synchrony between activity of two or more brain regions over a period of “rest” (i.e., a task-free state).
Triple network model of psychopathy: Framework describing multiple neuropsychiatric conditions in terms of dysregulated interactions between three large-scale brain networks (DMN, ECN, SN).
Error awareness: Ability to explicitly recognize and acknowledge one’s own mistakes when performing a task.
Error awareness task (EAT): Computerized paradigm assessing error awareness and error-related brain responsivity in which participants try to inhibit button-press responses and acknowledge their mistakes.

## Supporting Information

https://github.com/Flanneryg3/HIVCB_ProjectCode
