# Supplementary material for: Altered large-scale brain network interactions associated with HIV infection and error processing
Source: Netw Neurosci. 2022 Jul 1;6(3):791–815. doi: 10.1162/netn_a_00241 (PMC9810366; doi:10.1162/netn_a_00241)
Supplement: Supplementary file 1 [file netn-06-791-s001.docx]

**SUPPLEMENTAL INFORMATION**

**Altered large-scale brain network interactions associated with HIV infection and error processing**

Jessica S. Flannery^1^, Michael C. Riedel^2^, Lauren D. Hill-Bowen^1^, Ranjita Poudel^1^, Katherine L. Bottenhorn^1^, Taylor Salo^1^, Angela R. Laird^2^, Raul Gonzalez^1^, and Matthew T. Sutherland^1^*

^1^ Department of Psychology, Florida International University, Miami, FL, USA

^2^ Department of Physics, Florida International University, Miami, FL, USA

***Correspondence:**

Matthew T. Sutherland, Ph.D.

[masuther@fiu.edu](mailto:masuther@fiu.edu)

**SUPPLEMENTAL CONTENT**

**TABLES**

- Table S1: Resting-state scan motion metrics by group (pp. 2)
- Table S2: Participant mental health status by group (pp. 2)
- Table S3: Participant plasma THC and metabolite levels by group (pp. 2)
- Table S4: Participant drug dependence status by group (pp. 2)
- Table S5: Mean past month and lifetime drug use by group (pp. 3)

**TEXT**

- ICA-based approach for calculating the resource allocation indexes (RAIs) (pp. 4)

**FIGURES**

- Figure S1. HIV-associated effects when using ICA-derived masks for RAI and rsFC metrics (pp. 5)
- Figure S2. Error awareness behavior was linked with ICA-derived SN-DMN rsFC strength (pp. 6)
- Figure S3. Follow-up analyses assessing relationships between network function and error awareness (pp. 7)
- Figure S4. Relationships between error-related network responsivity and error awareness (pp. 7)**SUPPLEMENTAL TABLES**

**Table S1. Resting-state scan motion metrics by group.**

|  | **All Participants**  *n*=93 | **HIV+/CB+**  *n*=28 | **HIV+/CB-**  *n*=26 | **HIV-/CB+**  *n*=22 | **HIV-/CB-**  *n*=17 | **Group Effects (*p*’s)** | | |
| --- | --- | --- | --- | --- | --- | --- | --- | --- |
|  |  |  |  |  |  | HIV | CB | HIV×CB |
| Mean framewise displacement | 0.12±0.04 | 0.12±0.05 | 0.12±0.04 | 0.12±0.04 | 0.13±0.04 | 0.9 | 0.4 | 0.98 |
| % volumes censored | 6.2±6.0 | 5.2±4.2 | 4.6±4.9 | 8.0±7.8 | 7.6±6.9 | 0.4 | 0.3 | 0.8 |

NOTE. Volumes with a framewise displacement (FD) greater than 0.35mm were censored along with timepoints immediately preceding and following. Group effects were assessed with a HIV×CB ANOVA.

**Table S2.** **Participant mental health status by group.**

|  | **All Participants**  *n*=93 | **HIV+/CB+**  *n*=28 | **HIV+/CB-**  *n*=26 | **HIV-/CB+**  *n*=22 | **HIV-/CB-**  *n*=17 | **Group Effects (*p*’s)** | |
| --- | --- | --- | --- | --- | --- | --- | --- |
|  |  |  |  |  |  | HIV | CB |
| Major Depressive Episode (current) | 10 | 4 | 3 | 1 | 2 | 0.4 | 0.8 |
| Major Depressive Episode (past) | 22 | 11 | 3 | 4 | 4 | 0.5 | 0.1 |

NOTE. Mental health status is reported as count of participants meeting criteria for a current or past major depressive episode as assessed via the Structured Clinical Interview for DSM-5 Research Version (SCID-5-RV). Participants were excluded from the sample if they presented severe mental illnesses with psychotic or paranoid symptoms. Group effects were assessed with two Chi-square tests, one comparing the HIV+ versus HIV- groups and the other comparing the CB+ versus CB- groups.

**Table S3.** **Participant plasma THC and metabolite levels by group.**

|  | **All CB+ Participants**  *n*=50 | **HIV+/CB+**  *n*=28 | **HIV-/CB+**  *n* =22 | **Group Effects (*p*’s)** |
| --- | --- | --- | --- | --- |
| 9-carboxy-THC (ng/mL) | 761.9 (1,145.5) | 964.6 (1,403.4) | 503.9 (636.4) | 0.1 |
| THC/creatinine ratio (ng/mg) | 599.5 (690.8) | 622.0 (728.0) | 566.5 (650.1) | 0.8 |

NOTE. Data are expressed as mean (standard deviation). Group effects were assessed with an independent samples *t*-test comparing cannabis using participants that were HIV+ versus HIV-.

**Table S4. Participant drug dependence status by group (count).**

|  | **All Participants**  *n*=93 | **HIV+/CB+**  *n*=28 | **HIV+/CB-**  *n*=26 | **HIV-/CB+**  *n*=22 | **HIV-/CB-**  *n*=17 | **Group Effects (*p*’s)** | | | |
| --- | --- | --- | --- | --- | --- | --- | --- | --- | --- |
|  |  |  |  |  |  | HIV | CB | | |
| Cannabis (past month) | 10 | 6 | 0 | 4 | 0 | 0.9 | **0.002** | | |
| Cannabis (lifetime) | 17 | 8 | 0 | 9 | 0 | 0.4 | | **<0.001** | |
| Alcohol (lifetime) | 10 | 3 | 2 | 3 | 2 | 0.7 | | | 0.7 |
| Cocaine (lifetime) | 8 | 3 | 1 | 3 | 1 | 0.7 | | | 0.3 |
| Other stimulants (lifetime) | 3 | 3 | 0 | 0 | 0 | - | | | - |
| Opiates (lifetime) | 1 | 1 | 0 | 0 | 0 | - | | | - |
| Other sedatives (lifetime) | 2 | 2 | 0 | 0 | 0 | - | | | - |
| Hallucinogens (lifetime) | 2 | 2 | 0 | 0 | 0 | - | | | - |
| Other drug (lifetime) | 2 | 1 | 0 | 1 | 0 | - | | | - |

NOTE. Drug dependence data are expressed as the count of participants meeting dependency criteria either ever (lifetime) or in the past month (past month), as defined in the DSM-5 Structured Clinical Interview. As current (past month) drug dependence was exclusionary criteria (except cannabis dependence in the CB+ groups), no participants met dependency criteria in the past month for any of the reported drugs. Group effects were assessed with two Chi-square tests, one comparing the HIV+ vs. HIV- groups and one comparing the CB+ vs. CB- groups. These analyses were not performed when data did not meet criteria for Chi-square tests (cells with <5 observations).

**Table S5. Mean past month and lifetime participant drug use by group (times used).**

|  | **All Participants**  *n*=93 | **HIV+/CB+**  *n*=28 | **HIV+/CB-**  *n*=26 | **HIV-/CB+**  *n*=22 | **HIV-/CB-**  *n*=17 | **Group Effects (*p*’s)** | | |
| --- | --- | --- | --- | --- | --- | --- | --- | --- |
|  |  |  |  |  |  | HIV×CB | HIV | CB |
| Cannabis (past month) | 12.8 (14.0) | 23.4 (9.8) | 0 | 24.4 (10.1) | 0 | 0.7 | 0.7 | **<0.001*** |
| Cannabis (lifetime) | 2,140.7 (3,111.6) | 3,556.8 (2968.6) | 282.6 (1024.1) | 4,182.5 (3,852.1) | 7.8 (23.7) | 0.4 | 0.7 | **<0.001*** |
| Alcohol (past month) | 2.2 (3.3) | 2.2 (2.6) | 2.2 (4.2) | 2.9 (3.2) | 1.4 (2.9) | 0.3 | 0.9 | 0.3 |
| Alcohol (lifetime) | 1,110.9 (1,557.3) | 1,128.1 (1,498.8) | 1,314.0 (1,918.0) | 1,369.4 (1,574.5) | 437.2 (714.9) | 0.1 | 0.3 | 0.3 |
| Cocaine (past month) | 0.02 (0.1) | 0.1 (0.3) | 0 | 0 | 0 | 0.2 | 0.2 | 0.2 |
| Cocaine (lifetime) | 268.8 (917.9) | 260.5 (836.0) | 430.7 (1,267.3) | 289.0 (890.0) | 8.9 (29.4) | 0.3 | 0.3 | 0.8 |
| Nicotine (past month) | 4.3 (10.2) | 7.2 (12.7) | 1.5 (6.0) | 7.2 (12.8) | 0 | 0.7 | 0.7 | **0.002*** |
| Nicotine (lifetime) | 1614.0 (3,205.1) | 1,965.0 (2,805.6) | 1,575.0 (4,198.9) | 2,327.1 (3,351.5) | 172.6 (607.8) | 0.2 | 0.4 | 0.06 |
| Methamphetamine (past month) | 0.02 (0.2) | 0.1 (0.4) | 0 | 0 | 0 | 0.4 | 0.4 | 0.4 |
| Methamphetamine (lifetime) | 21.2 (126.9) | 60.2 (222.7) | 0 | 13.1 (61.4) | 0 | 0.4 | 0.4 | 0.2 |
| Prescription stimulants (past month) | 0.02 (0.2) | 0 | 0 | 0.1 (0.4) | 0 | 0.3 | 0.3 | 0.3 |
| Prescription stimulants (lifetime) | 25.5 (224.2) | 80.0 (407.8) | 0 | 6.0 (25.5) | 0 | 0.4 | 0.4 | 0.4 |
| Heroin (past month) | 0 | 0 | 0 | 0 | 0 | - | - | - |
| Heroin (lifetime) | 0.01 (0.1) | 0 | 0 | 0.05 (0.2) | 0 | 0.3 | 0.3 | 0.3 |
| Opiates (past month) | 0 | 0 | 0 | 0 | 0 | - | - | - |
| Opiates (lifetime) | 13.1 (83.2) | 29.5 (135.8) | 0 | 17.2 (76.7) | 0.7 (2.9) | 0.7 | 0.7 | 0.2 |
| Benzodiazepines (past month) | 0.01 (0.1) | 0 | 0 | 0.04 (0.2) | 0 | 0.3 | 0.3 | 0.3 |
| Benzodiazepines (lifetime) | 39.5 (273.4) | 128.8 (492.8) | 0 | 2.6 (6.5) | 0 | 0.3 | 0.3 | 0.3 |
| Barbiturates (past month) | 0 | 0 | 0 | 0 | 0 | - | - | - |
| Barbiturates (lifetime) | 7.6 (51.8) | 11.6 (61.2) | 0 | 0.04 (0.2) | 0 | 0.9 | 0.2 | 0.9 |
| Ecstasy (past month) | 0 | 0 | 0 | 0 | 0 | - | - | - |
| Ecstasy (lifetime) | 32.1 (100.6) | 67.1 (156.9) | 0.3 (1.6) | 49.5 (93.1) | 0.5 (1.5) | 0.7 | 0.7 | **0.006*** |
| Inhalants (past month) | 0.02 (0.2) | 0.1 (0.4) | 0 | 0 | 0 | 0.4 | 0.4 | 0.4 |
| Inhalants (lifetime) | 13.8 (77.8) | 43.1 (138.6) | 14.8 (75.3) | 3.2 (13.8) | 0 | 0.2 | 0.2 | 0.2 |
| Ever injection drug use (count) | 1 | 1 | 0 | 0 | 0 | - | *-* | *-* |

NOTE. Data are expressed as mean (standard deviation). Drug use was self-reported as the number of “times” using each drug in the given timeframe (past month or lifetime) on the National Survey on Drug Use and Health. We note that the CB using groups had significantly more lifetime ecstasy use than the non-using groups. However, as no participant had any recent ecstasy use (last month), we did not expect lifetime use differences to significantly impact our group effects of interest. Group effects were assessed with either an HIV×CB ANOVA or Chi square tests. * *p* < 0.05.

**ICA-based approach for calculating the resource allocation indexes (RAIs).**

To better control the spatial coverage of networks across participants we employed the atlas-based approach, described in the main text, when calculating the resource allocation indexes (RAIs) rather than an independent component analysis (ICA)-based approach which has been used in prior work. The lack of between-participant variability in network definition, allowed for the results from our group comparisons to be interpreted as differences in rsFC strength rather than differences in spatial network topology. Additionally, as we had *a priori* hypotheses regarding regions that may be impacted by HIV based on prior work implicating anterior insula and supplementary motor area regions of the salience network (SN) and the middle medial prefrontal cortex (mPFC), posterior cingulate cortex (PCC), and hippocampus regions of the default mode network (DMN), we wanted to ensure that our network masks included these regions. Thus, we used the FIND Lab’s anterior (anterior insula / dorsal ACC) SN subnetwork mask and dorsal (PCC/mPFC) DMN subnetwork. However, as prior work calculating RAIs has employed an ICA-based approach to identify networks and estimate participant-specific timeseries, we also employed an ICA-based approach, calculating our RAIs using networks identified via a group-average ICA (GICA). We generally arrived at the same outcomes and interpretations as described in the main text. Participants’ preprocessed function data was entered into a GICA using MELODIC’s temporal concatenation decomposition (FSL v.0.6.4) with a model order of 20.


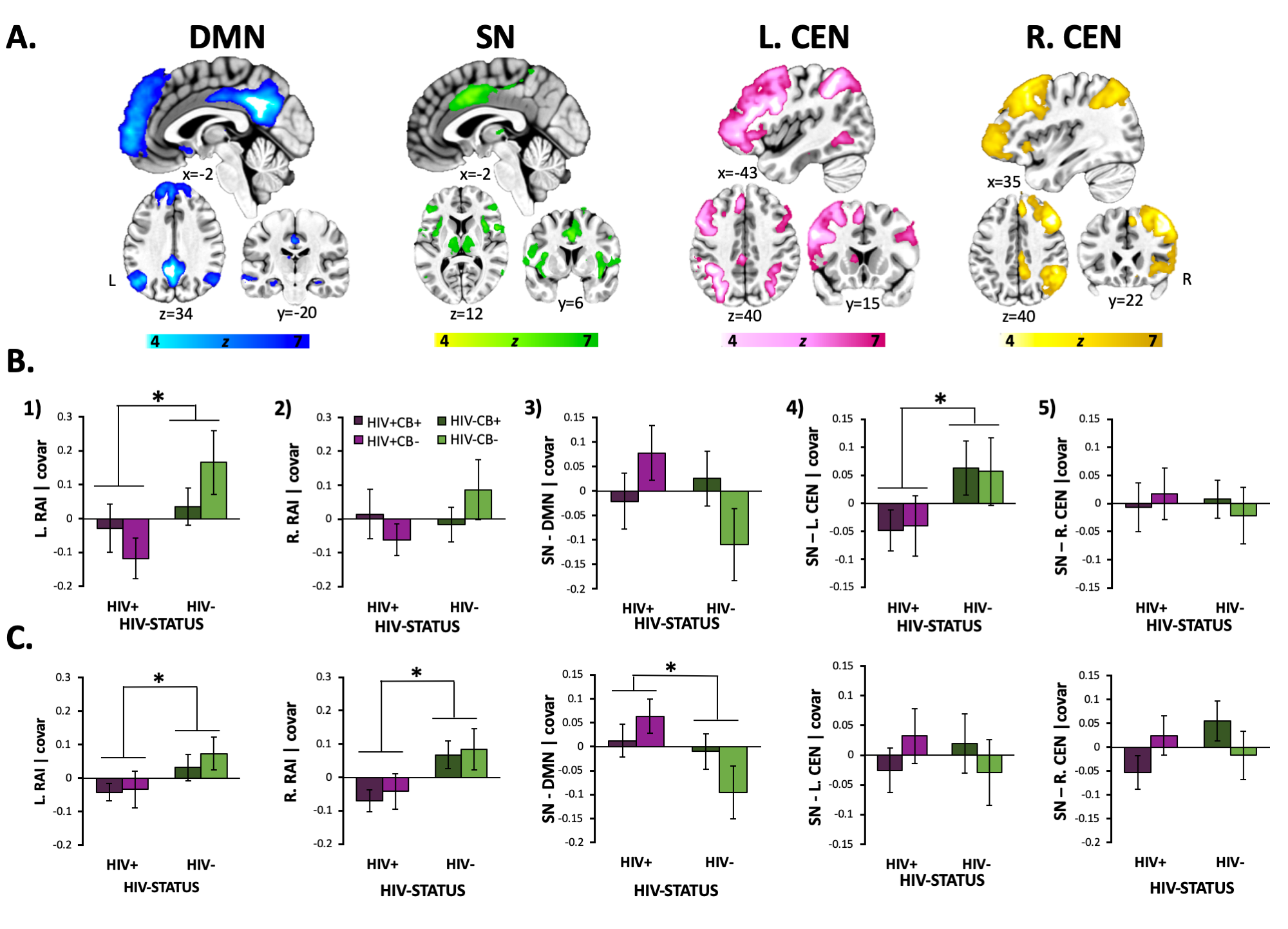


**Figure S1. HIV-associated effects when using ICA-derived masks for (RAI) and rsFC (SN-DMN, SN-CEN) metrics.**

**(A)** The resulting thresholded GICA spatial maps that corresponded to the SN, DMN, left central executive network (L.CEN), and right CEN (R.CEN) were identified by visual inspection. GICA component maps of interest were then regressed on each participant’s functional data as spatial predictors, to generate participant-specific timeseries for each network using dual regression (FSL v.0.6.4). These network timeseries were then used to calculate RAI values. Standardized correlation coefficients (CCs) between these ICA-derived network timeseries and RAIs were computed using the same approach as was applied for the atlas-based timeseries. RAIs derived via the ICA-based method were highly correlated with those derived with the atlas-based method (right: *p*<0.0001, left: *p*=0.017), albeit displayed more variability across subjects (standard deviation: ICA-based=0.36 [left], 0.33 [right] vs. atlas-based=0.24 [left], 0.22 [right]).

(**B1**) We assessed HIV×CB main and interaction effects with general linear models (GLM) including age, sex, mean framewise displacement (FD), and current cigarette smoking (NIC) status included as covariates. These GLMs were equivalent to the analyses run on RAIs and network resting-state functional connectivity (rsFC) measures derived from atlas-based approach described in the main text. PLWH (vs. HIV- participants) showed significantly reduced RAI values when considering the left hemisphere (HIV main effect: F[*1, 92*]= 5.9, *p*=0.017). (**B2**) However, unlike RAI values computed via the atlas-based approach, group effects did not reach significance when considering the right hemisphere (F[*1, 92*]=0.4, *p*=0.2). (**B3**) While, visual inspection suggests a similar direction of HIV and CB group effects on rsFC between the SN and DMN as those observed when considering atlas-derived measures, group effects did not reach significance (*p*’s>0.3). (**B4)** Interestingly, PLWH displayed significantly reduced rsFC between the SN and L.CEN compared to HIV- participants (F[*1, 92*]=4.2, *p*=0.04) while this same effect was not observed in the atlas-based approach. (**B5**) We did not observe any significant group-effects when considering rsFC between SN and R.CEN (*p*’s>0.8).

**(C)** Group-effects on atlas-derived network-level RAI and rsFC measures reproduced for comparison. Unstandardized residuals after regressing out covariates are plotted. Error bars = standard error of the mean.


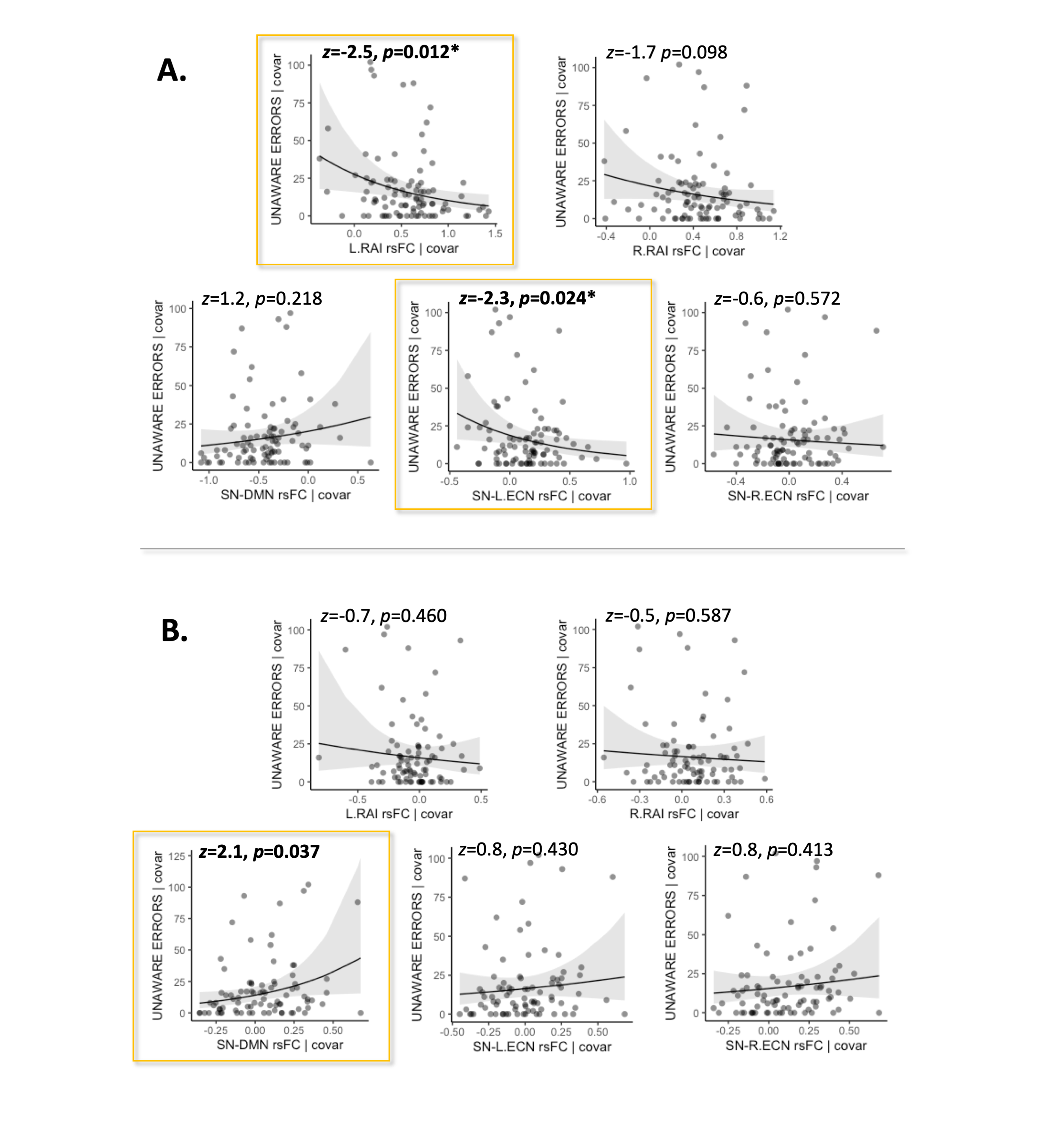


**Figure S2.** **Error awareness behavior was linked with ICA-derived SN-DMN rsFC strength.** **(A)** Relationships between frequency of unaware errors (error awareness), ICA-derived RAI values, and network rsFC strength. A negative binomial model (R, v.4.0.2) was used to statistically test these relationships as the error awareness variable was a positively skewed count variable and thus, did not meet assumptions required for linear regression. A negative binomial model was selected over a zero-inflated or Poisson model as the test of dispersion indicated over-dispersion. Age, sex, mean FD, and cigarette smoking (NIC) status were included as covariates in these analyses. Decreased left RAI values (*z*=-2.5, *p*=0.012) and SN-L.CEN rsFC (*z*=-2.3, *p*=0.024), were significantly associated with increased unaware errors. Contrarily, right RAI values (*z*=-1.7, *p*=0.098), SN-DMN rsFC (*z*=1.2, *p*=0.218), and SN-R.CEN rsFC (*z*=0.6, *p*=0.572) were not significantly associated with error awareness. **(B)** Relationships between error awareness and atlas-derived RAIs and network rsFC strength reproduced for comparison. Unstandardized residuals after regressing out effects of covariates are plotted.


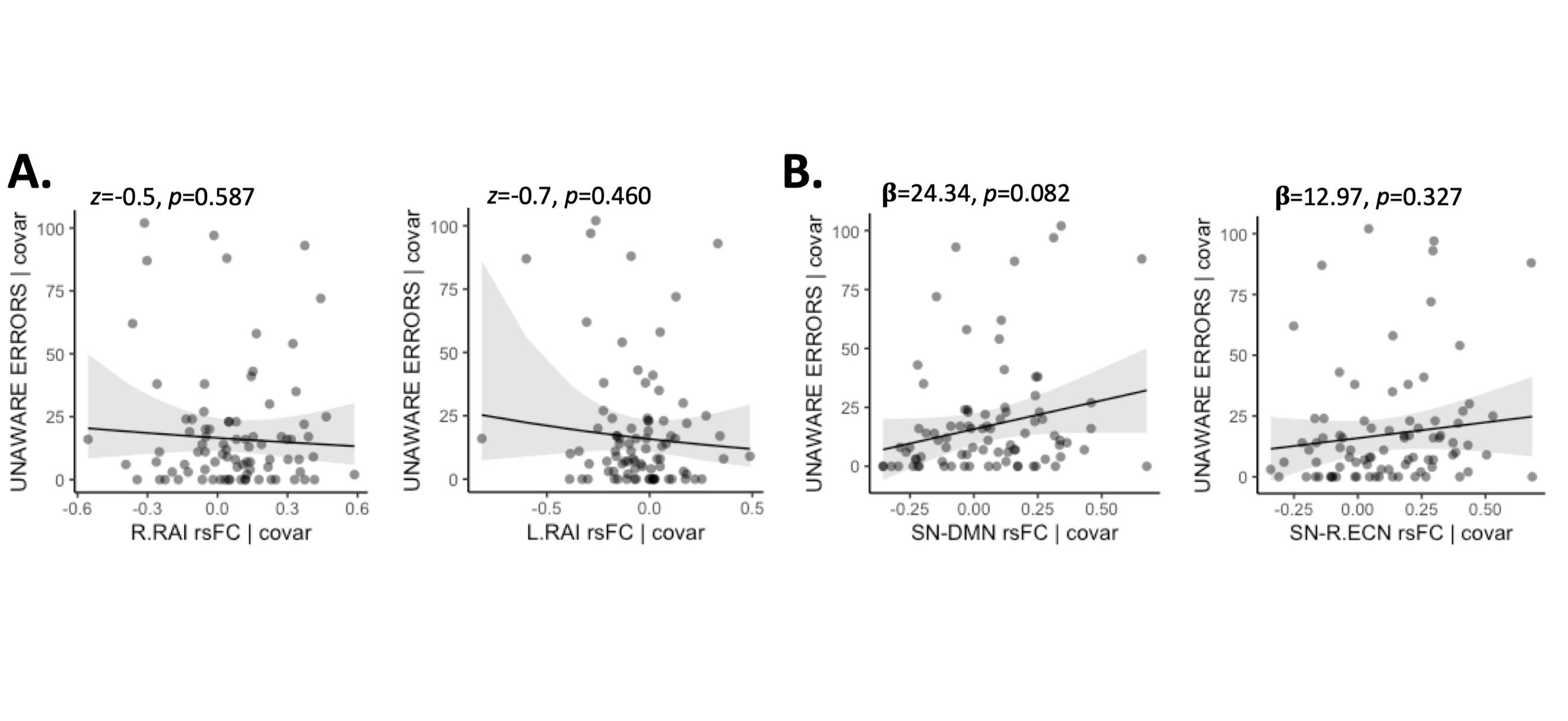


**Figure S3**. **Follow-up analyses assessing relationships between network function and error awareness**. (**A)** We employed a negative binomial model (R, v.4.0.2) including age, sex, mean FD, and NIC status as covariates to assess relations between left and right resource allocation index (RAI) values and error awareness (*n*=92). Neither left (*b*=-0.6 [0.8], *z*=-0.7, *p*=0.460) or right RAI values (*b*=-0.37 [0.7], *z*=-0.5, *p*=0.587) were significantly related to error awareness. **(B)** As the variable: frequency of unaware errors did not meet assumptions required for linear regression [33], a negative binomial model was used for analyses assessing the relationship between rsFC and unaware errors that are reported in the main text (**Figure 2**). Given the severe non-normality of the unaware error variable, we believe that the negative binomial model is the proper model for this analysis however, to increase transparency, we also re-ran this analysis employing a general linear regression while controlling for the same covariates (age, sex, mean FD, and NIC status). The relationship between SN-DMN rsFC and unaware errors no longer reached significance (*p*=0.082). Unstandardized residuals are plotted after regressing out effects of covariates.


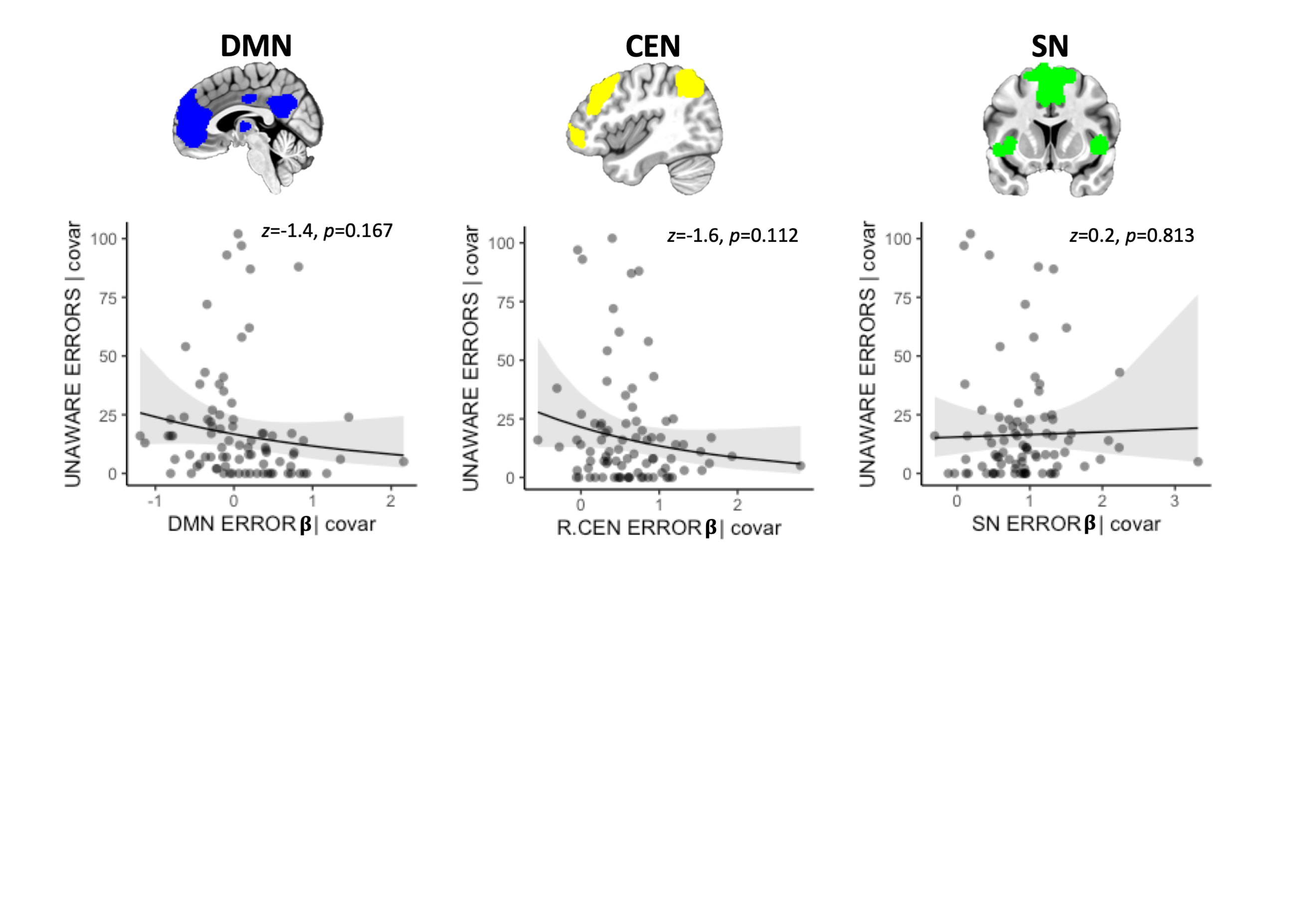


**Figure S4. Relationships between error-related network responsivity and error awareness.** Error-related DMN (*z*=-1.4, *p*=0.167), R. CEN (*z*=-1.6, *p*=0.112), L. CEN (data not shown: *z*=-1.4, *p*=0.169), and SN (*z*=0.2, *p*=0.813) responsivity in the error awareness task (EAT) was not significantly associated with error awareness, while controlling for sex, age, and cigarette smoking (NIC) status. Unstandardized residuals are plotted after regressing out effects of covariates. Error bars = standard error of the mean.
